# Thermal Evaluation of Multi-Antenna Systems Proposed to Treat Bone Tumors: Finite Element Analysis

**DOI:** 10.3390/s22197604

**Published:** 2022-10-07

**Authors:** Citlalli Jessica Trujillo-Romero, Juan Dionisio Merida, Texar Javier Ramírez-Guzmán, Raquel Martínez-Valdez, Lorenzo Leija-Salas, Arturo Vera-Hernández, Genaro Rico-Martínez, José Jesús Agustín Flores-Cuautle, Josefina Gutiérrez-Martínez, Emilio Sacristán-Rock

**Affiliations:** 1 Division of Medical Engineering Research, National Institute of Rehabilitation-LGII, Calz. México Xochimilco No. 289, Arenal de Guadalupe, Mexico City 14389, Mexico; 2 Department of Electrical Engineering, Universidad Autonoma Metropolitana, UAM-Iztapalapa, Av. Ferrocarril San Rafael Atlixco, 186, Leyes de Reforma, Mexico City 09310, Mexico; 3 Electrical Engineering Department, Bioelectronics Section, CINVESTAV-IPN, Instituto Politécnico Nacional 2508, San Pedro Zacatenco, Mexico City 07360, Mexico; 4 Biomedical Engineering Program, Universidad Politécnica de Chiapas, Suchiapa 29150, Mexico; 5 Bone Tumors Service, National Institute of Rehabilitation-LGII, Calz. México Xochimilco No. 289, Arenal de Guaudalupe, Mexico City 14389, Mexico; 6 CONACYT-National Technological Institute of Mexico/I.T. Orizaba, Posgraduate Studies and Research Division, Oriente 9, No. 852, Orizaba 94320, Mexico; 7 National Center for Research in Instrumentation and Medical Imaging, UAM-Iztapalapa, Av. Ferrocarril San Rafael Atlixco, 186, Leyes de Reforma, Mexico City 09310, Mexico

**Keywords:** microwave ablation, bone tumors, thermal ablation, antenna array, FEM modeling

## Abstract

Microwave ablation is commonly used in soft tissue tumors, but its application in bone tumors has been barely analyzed. Antennas to treat bone tissue (~3 cm^2^), has been lately designed. Bone tumors at pathological stage T1 can reach 8 cm wide. An antenna cannot cover it; therefore, our goal is to evaluate the thermal performance of multi-antenna arrays. Linear, triangular, and square configurations of double slot (DS) and monopole (MTM) antennas were evaluated. A parametric study (finite element method), with variations in distance between antennas (ad) and bone thickness (bt) was implemented. Array feasibility was evaluated by SWR, ablated tissue volume, etc. The linear configuration with DS and MTM antennas showed SWR ≤ 1.6 for ad = 1 mm–15 mm and bt = 20 mm–40 mm, and ad = 10 mm–15 mm and bt = 25 mm–40 mm, respectively; the triangular showed SWR ≤ 1.5 for ad = 5 mm–15 mm and bt = 20 mm–40 mm and ad = 10 mm–15 mm and bt = 25 mm–40 mm. The square configuration (DS) generated SWR ≤ 1.5 for ad = 5 mm–20 mm and bt = 20 mm–40 mm, and the MTM, SWR ≤ 1.5 with ad = 10 mm and bt = 25 mm–40 mm. Ablated tissue was 4.65 cm^3^–10.46 cm^3^ after 5 min. According to treatment time and array configuration, maximum temperature and ablated tissue is modified. Bone tumors >3 cm^3^ can be treated by these antenna-arrays.

## 1. Introduction

The most common procedures to treat bone cancer are surgery, radiotherapy, and chemotherapy. The chosen therapy depends on the condition and necessities of the patient; however, it is well-known that radiotherapy and chemotherapy are not totally effective. In radiotherapy, high doses are required due to the fairly low radiosensitivity of the bone; therefore, healthy tissue can be damaged [1]. Chemotherapy is less used because most of the bone tumors are not very sensitive to it [2]. Moreover, both therapies have the disadvantage of producing several side effects that reduce the patient quality of life. In recent years, thermotherapies such as thermal ablation have been proposed to treat bone tumors. Microwave ablation (MWA) is a minimally invasive technique where critical temperatures around 60 °C are reached as an effect of the deposition of electromagnetic (EM) energy [3]. Specifically, MWA is more effective in treating bone tumors because the tissue heats faster, and larger ablation zones are achieved [4,5,6]. Several authors report that MWA has been successfully used as a complementary treatment for different musculoskeletal tumors [7,8]. Microwave heating applicators work at frequencies in the ISM band (Industrial Scientific and Medical). One of the frequencies, 2.45 GHz, is the most used worldwide in medical practice for thermal ablation. The reported antennas work at 2.45 GHz and reach temperatures higher than 60 °C [9,10,11] when using more than 50 W as input power [4]. Although this means that thermal ablation is present, high input power levels are necessary because the tested antennas were designed to treat soft tissue instead of bone tissue. To compensate the power loss, the required treatment time and input power level to generate thermal ablation increases. 

Lately, researchers at the National Institute of Rehabilitation Luis Guillermo Ibarra Ibarra (INR-LGII) started developing micro-coaxial antennas that were designed to specifically treat bone tissue by MWA [12,13,14,15,16,17]. Trujillo-Romero et al. firstly designed a single slot antenna to treat bone tumors [15]. A double slot (DS) antenna was also designed for the same purpose; it was modeled, built, and characterized in ex vivo porcine bone. The results showed ablation temperatures (60–100 °C) are reached by applying 10 W per 10 min; moreover, the maximum standing wave ratio (SWR) was 1.8 [16]. Although both antennas were effective in treating bone tissue, their diameter (1.19 mm) allows its deformation. This effect could reduce the antenna lifetime and affect its performance after just a few uses. Therefore, a new set of four different micro-coaxial antennas with a thicker diameter (2.19 mm) was proposed [17]. The antennas were modeled, built, and tested by using phantoms and ex vivo porcine tissue. The antennas showed SWR values lower than 1.7; moreover, just 5 W were applied during 5 min to reach ablation temperatures (60–100 °C). The reduction of input power levels had been possible due to the optimization process of our antennas This process allows to improve the efficiency of the MW system; therefore, ablated temperatures are reached with lower levels of input power (less than 10 W) in a relatively short period of time (minutes). The proposed antennas can cover a tissue region of approximately 3 cm^2^. However, according to the pathological stage of cancer, bone tumors at stage T1 can reach no more than 8 cm, while at T2 can be greater than 8 cm wide [18]. This region dimension cannot be covered by using a single micro-coaxial antenna, then, either multiple antenna insertions or a multi-antenna system must be used. The literature reports that some studies were carried out to evaluate multi-antenna arrays; however, most of these studies were implemented in liver tissue [19,20,21,22]. Karampatzakis et. al. reported a study that was performed to evaluate the heating characteristics of antenna arrays by using computational modeling [23]. However, in this study the use of triangular and square configurations of double slot (DS) antenna arrays to treat liver tumors was proposed; additionally, a brief analysis of some other tissues, including bone, were also implemented. Furthermore, to our knowledge, our study is the first one completely focused on evaluating the effect of using multi-antenna arrays to treat bone cancer. Therefore, the goal of this work was to evaluate the thermal behavior of different micro-coaxial antenna arrays that were proposed to treat bone cancer by MWA. Our hypothesis is that by using micro-coaxial antenna arrays, it is possible to increase the volume of the treated tumor; moreover, by using different antenna types and treatment time, different shapes of the ablation zones can be generated. The micro-coaxial antennas that were included in the study were the double slot (DS) and the monopole (MTM) antenna. A 3D parametric computational study that was based on the finite element method was proposed. The antenna arrays of two (linear), three (triangular), and four (square) antennas were analyzed. Several scenarios considering relevant parameters such as distance between the antennas (ad) and bone thickness (bt) were evaluated. In order to estimate the thermal effect over the tissues, SWR (standing wave ratio) values, maximum temperatures reached, the volume of tissue at the ablation temperatures, and the thermal distributions were analyzed.

## 2. Materials and Methods

**Figure 1:**
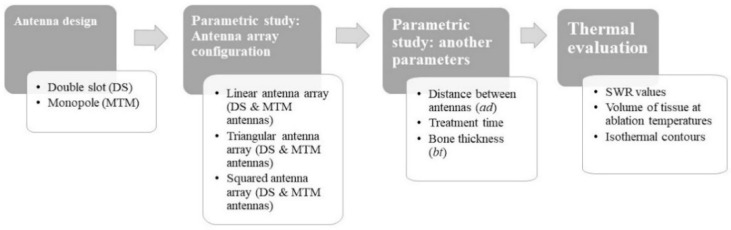
Step by step flow chart that was followed to implement the study.

Figure 1 shows a flow chart to summarize the implemented study step-by-step.

### 2.1. Micro Coaxial Antennas

**Figure 2:**
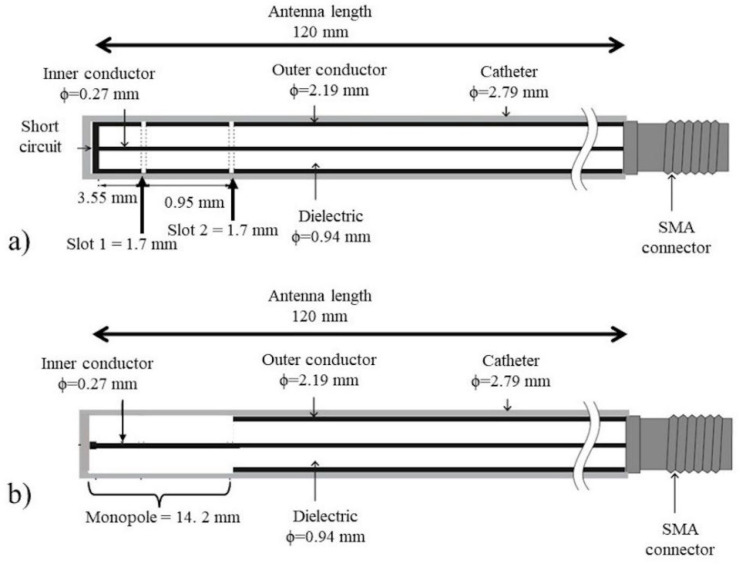
Proposed antennas, (**a**) double slot antenna and (**b**) monopole antenna.

To carry out this study, one of the most commonly used antennas to treat soft tissue (double slot antenna) was proposed. Moreover, as this research was focus on bone tumors, the monopole antenna was also proposed to evaluate the possibility to cover a larger region of bone tissue. The double slot and the monopole antennas were previously modeled, optimized, and tested in multi-tissue phantoms and ex vivo porcine tissue by this research group [17]. The computational models showed SWR values of 1.26 and 1.55 for the double slot (DS) and the monopole (MTM) antennas, respectively. Figure 2 shows the geometrical scheme of each antenna and its dimensions that ensure a maximum energy transmission. The antenna diameters were set in accordance with the geometric characteristics of the semi-rigid micro-coaxial cable UT-085; moreover, each antenna is placed in a catheter to avoid contact with the surrounding tissue. 

### 2.2. Finite Element Models (FEM) 

Electromagnetic and thermal models of the proposed multi-antenna systems were performed in COMSOL Multiphysics (COMSOL Inc., Burlington, MA, USA). The electromagnetic simulations were implemented in the frequency domain (2.45 GHz) and solved by using the stationary solver (used to find the solution to linear and nonlinear stationary problems). The thermal simulations were implemented as time-dependent by using the time-dependent solver (used to find the solution to time-dependent problems). 3D models that include the multi-antenna system as well as a multi-tissue segment (bone, muscle, fat, and skin) were implemented.

#### 2.2.1. Electromagnetic (EM) Models

To implement the FEM model and to predict the antenna’s performance, a current source (J_imp_) that irradiated an electromagnetic field was considered. Element Model Maxwell’s equations, described by Equations (1)–(4), govern the antennas’ performance [24].

(1) $$\nabla \times E=-j\omega \overset{↔}{\mu }\cdot H-M_{imp}\;$$

(2) $$\nabla \times H=-j\omega \overset{↔}{\varepsilon }\cdot E-J_{imp}$$

(3) $$\nabla \cdot \left(\overset{↔}{\varepsilon }\cdot E\right)=-\frac{1}{j\omega }\nabla \cdot J_{imp}$$

(4) $$\nabla \cdot \left(\overset{↔}{\mu }\cdot H\right)=-\frac{1}{j\omega }\nabla \cdot M_{imp}$$

where ***M****_imp_* is the magnetic current density, and $\overset{↔}{\varepsilon }$ and $\overset{↔}{\mu }$ are the permittivity and permeability of the tissues that were irradiated by the antenna, ***E*** is the electric field, and *H* is the magnetic field intensity. The boundary condition for the metallic surfaces, represented by Equation (5) is considered to solve them:

(5) $$n\times E=0\;r\;\epsilon {\;S}_{\mathrm{PEC}}$$

where SPEC represents the antenna body surface as a perfect electric conductor (PEC). Moreover, the feed point of each antenna was modeled as a port (micro coaxial) boundary condition. 

The E and H field satisfy the Sommerfeld radiation boundary condition. These equations can be hardly analytical solved; therefore, they are solved by numerical methods. Due to an EM field that is irradiated by the antenna to the infinite, the solution space must be limited (*S*_0_). Therefore, a new boundary condition to describe the EM waves propagation in a finite space is applied. The boundary condition, known as first-order absorbing, described by Equation (6), is applied to approximate this behavior. Moreover, this condition indicates that the EM field can travel in the space without reflections, i.e., the boundary does not disturb the EM field distribution.

(6) $$\hat{n}\times \nabla \times \left(\begin{array}{c} E\\ H \end{array}\right)+{\mathrm{jk}}_{0}\hat{n}\times \hat{n}\times \left(\begin{array}{c} E\\ H \end{array}\right)\;\approx 0\;r\epsilon S_{0\;}$$

where $\hat{n}$ is the unit vector normal to the surface *S*_0_. Equation (7) is obtained by doing all the mathematical procedures:

(7) $${∭}_{V}^{} [\left(\nabla \times T\right)\cdot \;{\overset{↔}{\mu_{r}}}^{-1}\;\cdot \left(\nabla \times E\right)-k_{0}^{2}T\cdot \;{\overset{↔}{\varepsilon }}_{r}\cdot E]dV\;\;={∯}_{S_{0\;}\cup \;S_{PEC}}^{}\hat{n}\cdot [T\times \left({\overset{↔}{\mu }}_{r}{}^{-1}\cdot \nabla \times E\right)]dS\;\;-{∭}_{V}^{}T\cdot [jk_{0}Z_{0}J_{imp}+\nabla \times \left({\overset{↔}{\mu }}_{r}{}^{-1}\cdot M_{imp}\right)]dV\;$$

where μ↔r =μ↔μ0 and ε↔r =ε↔ε0 are the relative permeability and permittivity tensor, respectively, $k_{0}=\omega \sqrt{\mu_{0}\varepsilon_{0}}$ is the wavenumber in free space, Z0=μ0ε0 is the intrinsic impedance, V is the volume confined by S_0_, and T the testing function.

To describe the EM problem, Equation (8) was obtained by applying the first-order absorbing boundary condition.

(8) $${∭}_{V}^{} [\left(\nabla \times T\right)\cdot {\overset{↔}{\mu }}_{r}{}^{-1}\cdot \left(\nabla \times E\right)-k_{0}^{2}T\cdot {\overset{↔}{\varepsilon }}_{r}\cdot E]d\;\;={∯}_{S_{PEC}}^{}\left(\hat{n}\times T\right)\cdot {\overset{↔}{\mu }}_{r}{}^{-1}\cdot \left(\nabla \times E\right)dS\;\;-jk_{0}\;{∯}_{S_{0}}^{}\left(\hat{n}\times T\right)\cdot \;\left(\hat{n}\times E\right)dS\;\;-{∭}_{V}^{}T\cdot [jk_{0}Z_{0}J_{imp}+\nabla \times \left({\overset{↔}{\mu }}_{r}{}^{-1}\cdot M_{imp}\right)]dV$$

To numerically solve the equation, V is divided in small finite elements.

A transverse electromagnetic field (TEM) describes the wave propagation in a micro-coaxial cable. Equations (9)–(11) describe the antenna wave propagation for a time-harmonic fields [25]:

(9) $$E=e_{r}\frac{C}{r}e^{j\left(\omega t-kz\right)},$$

(10) $$H=e_{\varphi }\frac{C}{rR}e^{j\left(\omega t-kz\right)},$$

(11) $$P_{av}=e_{z}\pi \frac{C^{2}}{Z}lnln\;\left(\frac{r_{inner}}{r_{outer}}\right)\;,$$

where z is the direction of propagation; *r*, *φ*, and *z* are the coordinates of the antenna body; *P_av_* is the power flow; *Z* is the wave impedance, *r_inner_*, and *r_outer_* are the radius of the inner and the outer conductors, respectively; ω is the angular frequency; and *k* the constant of propagation.

On the other hand, the specific absorption rate (SAR) describes the absorbed energy per unit of mass in a human body when it is exposed to an electromagnetic source, as described by Equation (12):

(12) $$SAR=\frac{\sigma }{2\rho }{\left|E\right|}^{2}\;$$

where *E* is the propagated electric field, σ and ρ are the conductivity (S/m), and density (kg/m^3^) of the irradiated medium, respectively. 

#### 2.2.2. Thermal Models

The thermal model is described by the Pennes bioheat transfer equation represented by Equation (13):

(13) $$\rho c\frac{\partial T}{\partial t}=\nabla \cdot \left(k\nabla T\right)+\rho Q+SAR-C_{b}W\left(T-T_{b}\right)$$

where *c* (J/kg/K) is the heat capacity, ρ (k/m^3^) is the tissue density, k (W/m ∗ K) is the thermal conductivity, *C_b_* is the blood heat capacity (J/Kg/K), W (kg/m^3^/s) is the blood perfusion, *T_b_* (*K*) is the blood temperature, *Q* (W/m^3^) is the metabolism heat generation, and SAR (W/kg) was previously described by Equation (12).

The FEM model represents the heat-transfer problem in a small section of the multi-tissue domain (bone, muscle, fat, and skin), i.e., the domains were truncated; therefore, an insulation boundary condition was applied as described by Equation (14):

(14) n·∇T=0 

where *T* is set as the initial physiological tissue temperature (37 °C). Table 1 presents the tissues and antenna properties that were used in the FEM modeling.

### 2.3. Parametric Study 

**Figure 3:**
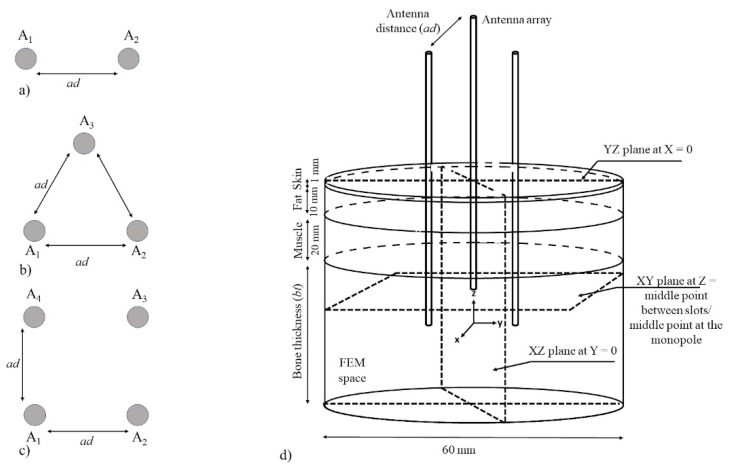
Proposed antenna array configurations to be analyzed, the antennas are depicted as A_1_ A_2_, A_3_, and A_4_ according to the antenna configuration, and example of the 3D computational model, (**a**) linear antenna array, (**b**) triangular antenna array, (**c**) square antenna array, and (**d**) example of the geometry that was used to implement this study. The geometry consists of a cylinder of multilayer tissue (bone, muscle, fat, and skin), as well as the evaluated antenna array. The thicknesses of muscle, fat, and skin were 2 cm, 1 cm, and 1 mm, respectively. The observation planes XY, XZ, and YZ that were used for the evaluation are also depicted. As previously described, the distance between antennas (*ad*) was modified from 1 mm to 15 mm and bone thickness (*bt*) from 2 to 4 cm.

Bone tumors can be more than 8 cm wide. To cover a more significant volume than the one that was covered by a single antenna, a study to evaluate the antenna arrays performance was proposed. Both antennas (DS and MTM) were evaluated by following the linear (Figure 3a), triangular (Figure 3b), and square (Figure 3c) antenna array configurations. In the study, each antenna was set as A_1_ A_2_, A_3_, and A_4_ according to the antenna configuration. A parametric study was performed to obtain the maximum distance between antennas (*ad*) that was needed to enhance the volume of tissue that was affected by the thermal ablation. In order to set the distance between antennas (*ad*), the next specifications, according to previous studies that were done by this research group, were considered [15,17]. The designed antennas reach temperatures higher than 60 °C at approximately 10 mm from the antenna axis, and similar temperatures were reached at approximately 6 mm further to the antenna tip. Therefore, the covered distance ad was set from 1 to 15 mm with steps of 5 mm. These distances were chosen to ensure that the thermal pattern of the antennas will be combined; consequently, larger volumes of tissue at ablation temperatures are expected. Moreover, to evaluate the maximum bone size that can be treated and the thermal effect over the other tissues (muscle, fat, and skin), the bone thickness (*bt*) was also included in the parametric study. Table 2 shows the values that were included in this study.

Figure 3d shows an example of a triangular configuration of antennas that was used to implement this study. Moreover, model dimensions, distance between antennas (*ad*), bone thickness (*bt*), and the axes of the observation planes that were used for the antenna performance evaluation, are represented.

### 2.4. Evaluated Parameters 

The standing wave ratio corresponding to each antenna was obtained. The SWR measures the impedance coupling among the load and the transmission line that supplies it. An SWR equal to 1 indicates that the entire power is transmitted to the irradiated medium. The higher the SWR, the greater power loss in the system; therefore, higher levels of power returns to the microwave system and damages either to the equipment or to the patient can be presented. On the other hand, maximum temperatures, tissue volumes at ablation temperatures, and temperature patterns that were generated by the antenna arrays were obtained. It is well-known that thermal damage depends on the reached temperature and the treatment time (energy exposure); temperatures around 50–55 °C reduce the necessary time to generate irreversible cell damage to 4–6 min. Temperatures around 60–100 °C must be reached to produce nearly immediate tissue and cell coagulation [27]. Therefore, in this study, the coagulation of tissue was considered once it reaches 55 °C. 

## 3. Results

### 3.1. Convergence Analysis

By using an adaptive mesh, a finer mesh in the region of interest was generated; therefore, the number of elements in the mesh can be reduced. An adequate mesh can help to reduce computational cost without an underestimation of the results. A convergence study was implemented to choose the adequate mesh size. Therefore, different mesh sizes were tested to perform the tests. The convergence test was done by analyzing the relationship between SWR and the simulation time versus the number of elements in the mesh. For all the antenna arrays, it was observed that the coarser mesh underestimates the results. For the linear arrays, models with around 625,885 (42 min)–842,209 (60 min) elements generated SWR values around 1.15. For triangular arrays, models with around 262,088 (30 min)–925,835 (80 min) generated stable SWR values around 1.34. Finally, for the square array, models with around 371,124 (60 min)–623,272 (130 min) elements generated SWR values around 1.4. It is important to address that due to the simplicity of the linear array models, the number of elements in the mesh can be increased without a big impact on the computational time.

### 3.2. Standing Wave Ratio (SWR)

**Figure 4:**
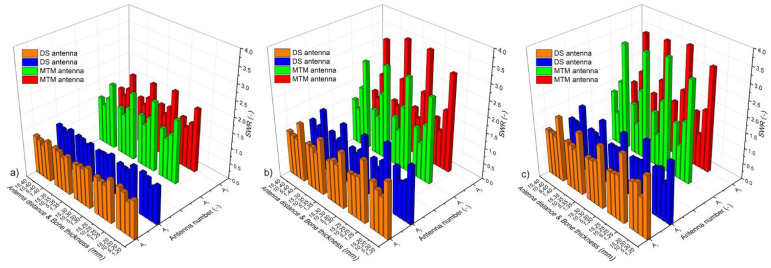
SWR of the antennas that compose the analyzed antenna arrays. (**a**) linear antenna array, (**b**) triangular antenna array (SWR for A_2_ is within the values of A_1_ and A_3_), and (**c**) square antenna array (SWR for A_2_ and A_4_ is within the values of A_1_ and A_3_). DS = double slot antenna, MTM = monopole antenna; A_1_, A_2_, A_3_, and A_4_ correspond to the antenna number in each antenna array.

Figure 4 shows the SWR values for linear, triangular, and square configurations of double slot (DS) and monopole (MTM) antennas. For triangular and square configurations, just the lowest and the highest SWR values (A_1_ and A_3_) are presented, i.e., the SWR values corresponding to antennas A_2_ and A_4_ were between the SWR values that are described in Figure 4. Figure 4a depicts the SWR for both antennas (A_1_ and A_2_) in the linear array that was composed of the DS and the MTM antennas. In all cases (antenna distance ad and bone thickness *bt*), the DS antennas showed better performance, i.e., the SWR was lower than 1.4, which means a better coupling of the antenna with the microwave system. On the other hand, the MTM antennas reached SWR values between 1.3 and 2, being the worst-case scenarios (SWR ≅ 2), the ones with *ad* = 1 mm, for all the analyzed bone thickness (*bt*). Figure 4b shows the SWR for the triangular array; the DS antennas generated SWR values that were lower than 1.5, except for those cases where *ad* = 1 mm. The MTM antennas with *ad* = 10 mm and *ad* = 15 mm had SWR values lower than 1.6; however, for *ad* = 1 mm and *ad* = 5 mm, the SWR was between 2 and 3. Finally, Figure 4c shows the SWR for the square array; in this case, the DS antenna shows SWR values between 1.3 and 1.5, except for those cases where *ad* = 1 mm (SWR ≥ 2). The MTM array with *ad* = 10 mm and *ad* = 15 mm had SWR values that were lower than 1.6; however, for *ad* = 1 mm and *ad* = 5 mm, the SWR was between 2 and 3.7. Moreover, to use the MTM antenna in multi-antenna arrays, the *bt* must be higher than 20 mm.

### 3.3. Microwave Heat-Source Density and Thermal Distributions

**Figure 5:**
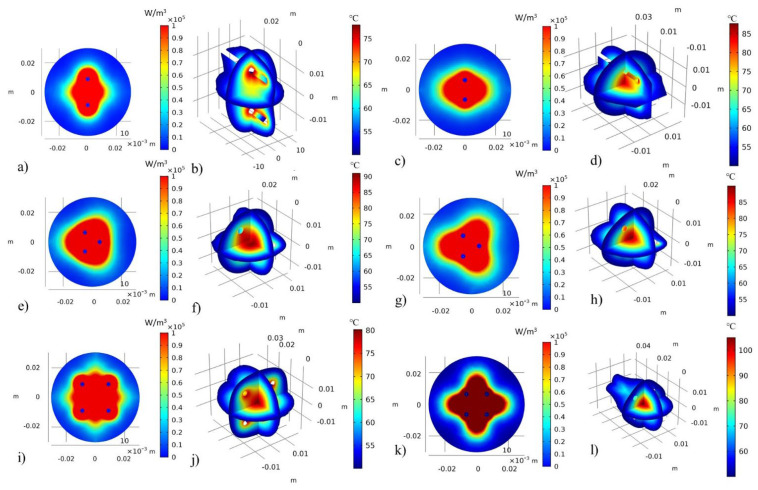
Distributions of the microwave heat source in the observation plane XY (W/cm^3^) and 3D views of the thermal distribution at temperatures higher than 50 °C. (**a**) heat source field for a linear array of doble slot antennas (*ad* = 15 mm and *bt* = 30 mm), (**b**) thermal distribution generated by the linear array of doble slot antennas *ad* = 15 mm and *bt* = 30 mm after 5 min, (**c**) heat source field for a linear array of monopole antennas (*ad* = 10 mm and *bt* = 40 mm), (**d**) thermal distribution generated by the linear array of monopole antennas (*ad* = 10 mm and *bt* = 40 mm) after 10 min, (**e**) heat source field for a triangular array of doble slot antennas (*ad* = 10 mm and *bt* = 40 mm), (**f**) thermal distribution generated by the triangular array of doble slot antennas (*ad* = 10 mm and *bt* = 40 mm) after 5 min, (**g**) heat source field for a triangular array of monopole antennas (*ad* = 10 mm and *bt* = 40 mm), (**h**) thermal distribution generated by the triangular array of monopole antennas (*ad* = 10 mm and *bt* = 40 mm) after 5 min, (**i**) heat source field for a square array of doble slot antennas (*ad* = 15 mm and *bt* = 40 mm), (**j**) thermal distribution generated by the square array of doble slot antennas (*ad* = 15 mm and *bt* = 40 mm) after 10 min, (**k**) heat source field for a square array of monopole antennas (*ad* = 10 mm and *bt* = 25 mm), (**l**) thermal distribution generated by the square array of monopole antennas (*ad* = 10 mm and *bt* = 25 mm) after 5 min.

Figure 5 shows the distributions of the microwave heat source in the observation plane XY, that was generated for the best-case scenarios, as well as the 3D views of the thermal distribution at temperatures higher than 50 °C. Clearly, the temperature field distributions are strongly related with the heat source distribution. Figure 5a,c,e,g,i,k shows how the microwave heat-source density can be modified as a function of the antenna type and antenna distance (ad). It is observed that the fields that are generated by each antenna are combined; therefore, the region of tissue that reaches thermal ablation is bigger. Consequently, the heat distributions are also different for each antenna array, as can be observed in Figure 5b,d,f,h,j,l. It is also observed how the reached temperature is different for each case-scenario. Moreover, the reached temperature also depends on the treatment time; for example, Figure 5b,d represent the thermal distribution after 10 min, Figure 5f,h after 5 min, while Figure 5j,l after 10 and 5 min, respectively. The reached temperatures depend on the antenna type, antenna distance (*ad*), and the treatment time.

### 3.4. Linear Antenna Array 

**Figure 6:**
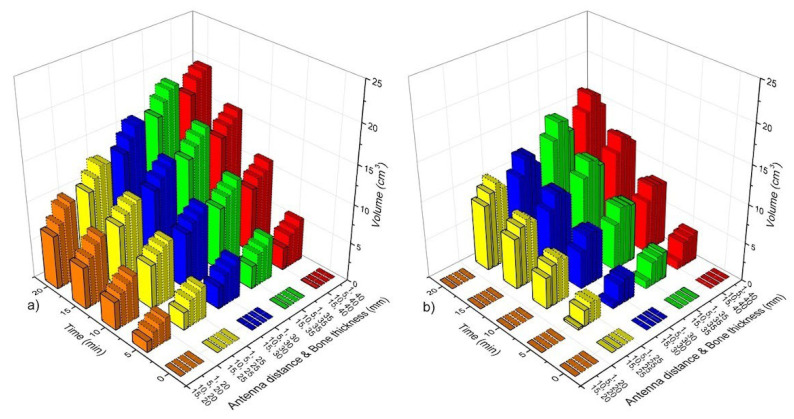
Bone tissue volume at ablation temperatures (T > 55 °C) as a function of time, antenna distance (*ad*), and bone thickness (*bt*). (**a**) Linear array of DS antennas, (**b**) linear array of MTM antennas.

Figure 6 shows the volume of bone tissue at ablation temperatures (T > 55 °C) as a function of time, antenna distance (*ad*), and bone thickness (*bt*). According to the SWR values (Figure 5a), all the case scenarios that were analyzed for the DS linear antenna array had a good performance (SWR < 1.4). Therefore, Figure 6a shows the volumes of bone tissue at ablation temperatures that were reached by the DS antenna array. As it was expected, the volume of heating tissue increases a function of time; moreover, the smaller the *ad*, the higher the tissue volume at ablation temperatures. Figure 6b shows the volume of bone tissue at ablation temperatures that was obtained with the linear array of MTM antennas. The best-case scenarios for each analyzed bone thickness (*bt*) were obtained with *ad* = 5 mm and *ad* = 10 mm. Although the rest of the cases for the MTM antenna arrays showed ablation temperatures in bone tissue, the SWR values for those antennas were approximately 2, which means higher power losses that can either overheat the patient or damage the equipment. Moreover, the tissue volumes that were reached by the MTM antenna array were lower than those that were reached with the DS antenna array. As it could be observed, different volumes of bone tissue at ablation temperatures could be reached depending on antenna type, treatment time, antenna distance, and bone thickness.

Table 3 shows the performance of the best-case scenarios for each bone thickness (*bt* = 20 mm–40 mm). The SWR, maximum temperature, and volume of bone (V__bone_) and muscle (V__muscle_) at ablation temperatures (T > 55 °C) are shown. For the DS antenna array, the best antenna distance was 5 mm; except for a *bt* = 30 mm, where *ad* = 15 mm was the best option. In all cases, ablation temperatures were reached after 5 min; however, the tissue volumes under ablation were lower than those that were reached after 20 min. Although the volume of bone tissue at ablation temperatures was between 9.74 cm^3^ and 19.15 cm^3^ after 20 min, the maximum reached temperatures were greater than 100 °C; moreover, the surrounding muscle tissue was also affected by temperature increases. It must consider that treatment time can be reduced from 20 min to 15 min to reduce the temperature and the effect on muscle. The MTM antenna showed maximum temperatures that were lower than 100 °C even after 20 min. The volume of bone at ablation temperatures goes from 9.81 cm^3^ to 16.49 cm^3^. After 20 min, the maximum muscle volume at ablation temperatures was 5.02 cm^3^, which means that muscle tissue is less affected when the MTM antenna is used. It is important to address that treatment time plays an important role in the volume of tissue (either bone or muscle) that reaches ablation.

**Figure 7:**
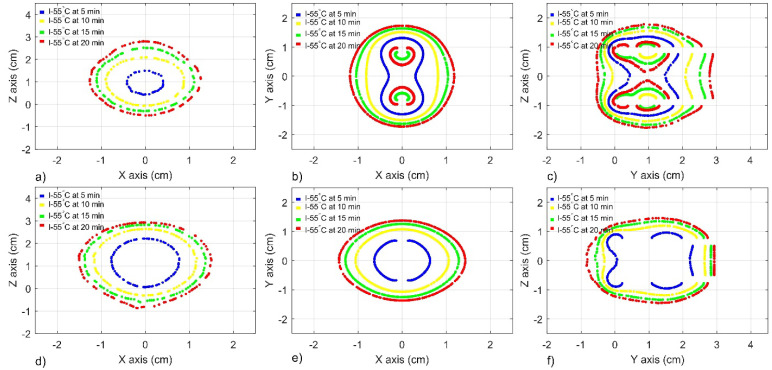
Isotherm contour at 55 °C generated by a linear antenna array over different planes after 5 min, 10 min, 15 min, and 20 min of treatment time. (**a**–**c**) XZ, XY, and YZ planes for a DS antenna array with ad = 15 mm and bt = 30 mm, respectively (**d**–**f**) XZ, XY, and YZ planes for a MTM antenna array with ad = 10 mm and bt = 40 mm, respectively.

Figure 7 shows the projections of the ablation zones on three observation planes, as explained below, for treatment times of 5, 10, 15, and 20 min. The isothermal contours at 55 °C that were generated by linear DS and MTM antenna arrays are presented. In these cases, each antenna was fed with 3 W, which means a total power of 6 W. The observation was throughout one transversal and two longitudinal planes. The transversal observation plane (XY) intersects the antenna insertion path at the height between both slots and at the middle point of the monopole. The longitudinal observation plane XZ and YZ crosses the centroid of the geometry of each configuration over the respective axes (See Figure 3). Figure 7a–c show the performance of a linear DS antenna array with *ad* = 15 mm and *bt* = 30 mm, at planes XZ, XY, and YZ, respectively; while Figure 7d–f show the performance of a linear MTM antenna array with *ad* = 10 mm and *bt* = 40 mm at the same planes. It was observed that even after 5 min of treatment time, both configurations were capable of generating continuous ablation zones, which became more extended as a function of treatment time. Both configurations generated different isothermal contours; however, it is observed that the longer the distance between antennas (*ad*), the smaller the ablation zone. Moreover, the DS antenna array shows temperatures higher than 100 °C in plane XY for times above 15 min and in plane YZ for times above 10 min, while the MTM antenna array does not generate overheating even after 20 min. Figure 6 shows how the isothermal contour can be modified by antenna type, antenna distance (*ad*), and bone thickness (*bt*).

Table 4 shows the ablation zone distances along the X, Y, and Z axes for 5 and 20 min. In order to quantify the uniformity of the ablation zone, a sphericity index was calculated. It was the ratio between the ablation zone volume when it is calculated as an ellipsoid by considering the distances (DX, DY, DZ), divided by the volume of a sphere that was generated by considering the longest distance. The distances (DX, DY, DZ) were calculated as the widest distance of the isothermal contour at 55 °C along the three axes. 

### 3.5. Triangular Antenna Array

**Figure 8:**
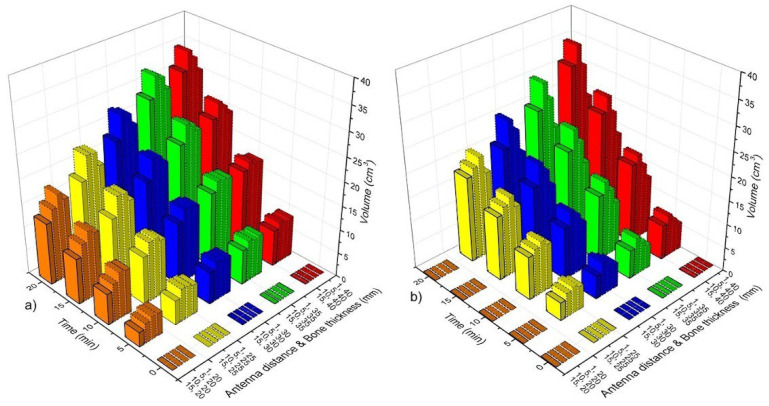
Bone tissue volume at ablation temperatures (T > 55 °C) as a function of time, antenna distance (*ad*), and bone thickness (*bt*). (**a**) Triangular array of DS antennas and (**b**) triangular array of MTM antennas.

Figure 8 shows the volume of bone tissue at ablation temperatures that were reached by the triangular antenna array. Figure 8a shows the volumes that were generated by the DS antenna array. In this case, and according to the SWR values (See Figure 4b), all cases, except the ones with *ad* = 1 mm and *bt* = 20 mm–40 mm (SWR ≅ 2), had a good performance, i.e., these cases present SWRs values <2 and V__bone_ ≅ 4 cm^3^ (5 min)–31 cm^3^ (20 min). The MTM antenna array with *ad* = 1 mm and *bt* = 25 mm–40 mm showed SWR values ≥ 3; however, for the rest of the cases, the SWRs <2 and V__bone_ ≅ 4 cm^3^ (5 min)–31 cm^3^ (20 min). Although the DS antenna array and the MTM antenna array reach similar V__bone_ after 20 min, in general, with the MTM antenna array, the V__bone_ achieved is lower than those achieved by the DS antenna array, as can be observed in Figure 8a,b. The triangular array, either DS or MTM antennas, produces a more significant temperature effect over the muscle tissue, which means that muscle can also reach ablation temperatures depending on treatment time.

Table 5 shows the best-case scenarios that were obtained with the triangular antenna arrays for each analyzed bone thickness (*bt*). The DS antenna array, *bt* = 20 mm and *ad* = 5 mm generated bone tissue overheating (T__max_ > 100 °C) even after 5 min of treatment time (T__max_ = 111.20 °C). Therefore, treatment times that are lower than 3 min are recommended; however, the ablated tissue volume will be less than 4 cm^3^. An increase in the antenna distance (*ad* = 10 mm) helps to reduce T__max_ and increase the volume of ablated bone. Moreover, if the treatment time increases, so does the tissue volume under ablation. However, not only bone but also muscle tissue reached ablation temperatures. After 5 min, the muscle volume (V__muscle_) at ablation temperatures could be considered within the safety margin. Nevertheless, after 20 min, the volume of the heated muscle increased considerably, especially for those cases with *bt* = 20 mm and 25 mm, where V__muscle_ = 13.55 cm^3^ and 10.11 cm^3^, respectively. Nevertheless, it will always depend on the tumor volume that is to be treated.

**Figure 9:**
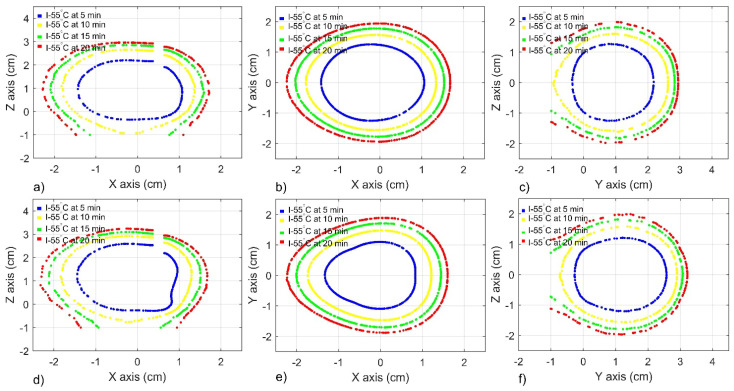
Isotherm contour at 55 °C generated by a triangular antenna array over different planes after 5 min, 10 min, 15 min, and 20 min of treatment time. (**a**–**c**) XZ, XY, and YZ planes for a DS antenna array with *ad* = 10 mm and *bt* = 40 mm, respectively (**d**–**f**) XZ, XY, and YZ planes for a MTM antenna array with *ad* = 10 mm and *bt* = 40 mm, respectively.

Figure 9a–c show the performance of a triangular DS antenna array with *ad* = 10 mm and *bt* = 40 mm, at planes XZ, XY, and YZ, respectively, while Figure 9d–f show the performance of a triangular MTM antenna array with *ad* = 10 mm and *bt* = 40 mm at the same planes. As in the linear antenna array, the triangular configurations generated continuous ablation zones, even after 5 min of treatment time. In this case, the best-case scenarios had the same *ad* (10 mm) and *bt* (40 mm). Therefore, both configurations generated quite similar isothermal contours, with slight differences (See Table 6). By combining *ad* and *bt* dimensions adequately, the reached temperatures did not surpass the 100 °C in any of the analyzed planes (See Table 5).

Table 6 shows the ablation zone distances along the X, Y, and Z axis and the sphericity index for 5 and 20 min generated by the triangular configurations of DS and MTM antennas. 

### 3.6. Square Antenna Array

**Figure 10:**
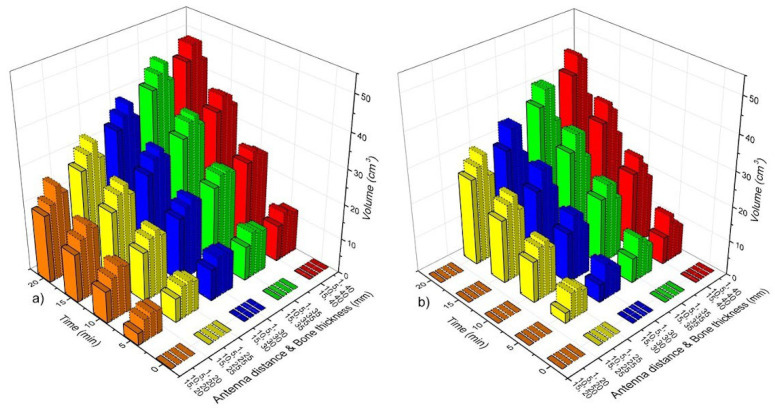
Bone tissue volume at ablation temperatures (T > 55 °C) as a function of time, antenna distance (*ad*), and bone thickness (*bt*). (**a**) Square array of DS antennas and (**b**) square array of MTM antennas.

Figure 10 shows the volume of bone tissue at ablation temperatures that were reached by the square configuration. Figure 10a shows the volumes that were generated by the DS antenna array. According to the SWR values (See Figure 4c), cases corresponding to *ad* = 1 mm and *bt* = 20 mm–40 mm had SWR values that were higher than 2, which means that their performance is not highly effective. Nevertheless, if ad increases, lower SWR values are achieved (See Table 7). Moreover, the tissue volume at ablation temperatures also increases. The volume of bone at ablation temperatures goes from approximately 7 cm^3^ (5 min) to 44.5 cm^3^ (20 min). Although the bone volume at ablation temperatures can be up to 44.5 cm^3^, the temperature increase also affects muscle and fatty tissues. In the worst-case scenario, even muscle was more affected than bone tissue, i.e., the V__bone_ = 19.5 cm^3^, V__muscle_ = 25.33 cm^3^, and V__fat_ = 2.23 cm^3^ for *ad* = 15 mm and *bt* = 20 mm. On the other hand, the MTM antenna array reached lower temperatures. Moreover, just cases where *ad* = 10 mm and 15 mm and *bt* = 25 mm–40 mm showed SWR values ≤2 (See Table 7). Therefore, in these cases, the reached volumes of bone were from 7 cm^3^ (5 min) to 41 cm^3^ (20 min). In the worst-case scenario (*ad* = 15 mm and *bt* = 20 mm), V__bone_ = 24.87 cm^3^, V__muscle_ = 19.5 cm^3^, and V__fat_ = 0.37 cm^3^.

Table 7 shows the best-case scenarios for each analyzed *bt* with a square configuration. For a square DS antenna array with *ad* = 5 mm and *bt* = 20 mm, tissue overheating was observed, and maximum temperatures of 132.3 °C and 139.9 °C were reached after 5 min and 20 min, respectively. Moreover, after 20 min, volumes of bone and muscle at ablation temperatures were quite similar (V__bone_ = 26.22 cm^3^ vs. V__muscle_ = 20 cm^3^); therefore, the focalization of the EM energy is not predominant over bone tissue. Nevertheless, the DS antennas array had the best performance with a maximum distance between the antennas (*ad* = 15 mm) and bone thickness higher than 25 mm (*bt* = 25 mm–40 mm). In these cases, the SWR values were lower than 1.5, and the reached temperatures did not overpass the ablation range (55–100 °C) even after 20 min. Although muscle may still be affected, in order to reduce its damage, the treatment time can be reduced. On the other hand, for the square MTM antenna, the achieved SWR values were the lowest (approx. 1.1); therefore, a maximum energy transmission was obtained; consequently, an overheating of tissue was reached, even after 5 min, where temperatures between 105 °C and 108 °C and a maximum energy focus over bone tissue were presented. At 20 min of treatment time, a maximum temperature of 140 °C was reached, and muscle tissue was highly affected by the temperature increase.

**Figure 11:**
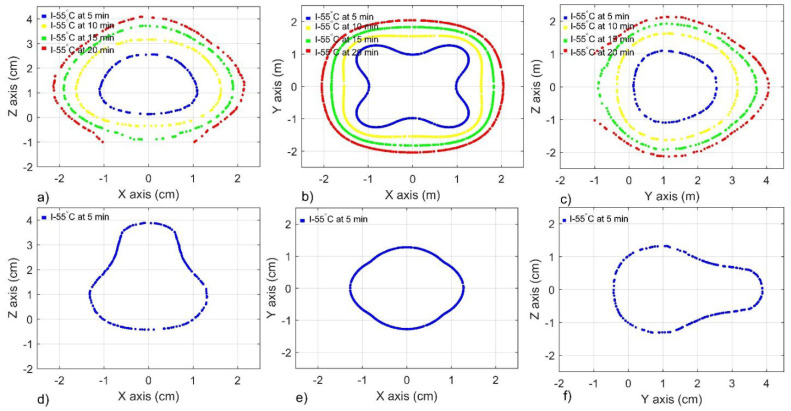
Isotherm contour at 55 °C that was generated by a square antenna array over different planes after 5 min, 10 min, 15 min, and 20 min of treatment time. (**a**–**c**) XZ, XY, and YZ planes for a DS antenna array with *ad* = 15 mm and *bt* = 40 mm, respectively; (**d**–**f**) XZ, XY, and YZ planes for a MTM antenna array with *ad* = 10 mm and *bt* = 25 mm, respectively.

Figure 11a–c show the performance of a square DS antenna array with *ad* = 15 mm and *bt* = 40 mm, at planes XZ, XY, and YZ, respectively, while Figure 11d–f show the performance of a square MTM antenna array with *ad* = 10 mm and *bt* = 25 mm at the same planes. After 5 min of treatment time, both configurations generated continuous ablation zones, which became more extensive as a function of treatment time. Both configurations generated different isothermal contours. Figure 10 shows how the isothermal contour can be modified in accordance with antenna type, antenna distance (*ad*), and bone thickness (*bt*). The fact that the total input power, in these cases, was 12 W must be addressed (3 W per antenna) because the reached temperatures were the highest from the study. Moreover, the MTM array generates maximum temperatures that are higher than 140 °C just after 5 min of treatment time. Therefore, in this case, just the isothermal contours at 5 min are shown in Figure 11.

Table 8 shows the ablation zone distances along the X, Y, and Z axis and the sphericity index for 5 and 20 min that was generated by the square configurations of DS and MTM antennas. For the MTM antenna, the sphericity index shows a reduction of the ablation zone uniformity; however, this configuration can still be used to treat different tumor shapes. The values for 20 min are not reported because the tissue is overheated (140 °C).

## 4. Discussion

Thermal ablation as bone tumors treatment has been poorly analyzed; just a few studies about it are reported in the literature. Moreover, in most of these cases, antennas that are designed to treat soft tissue have been used, making it necessary to use either a high input power or large treatment time. Recently, new micro-coaxial antennas specifically designed to treat bone tumors has been proposed by this research group. However, the first evaluations showed that to treat large bone tumors, multi-antenna arrays must be used. The main goal of this paper was to evaluate the performance of three array configurations (linear, triangular, and square) of antennas that were designed specially to treat bone tissue. The evaluation was based on SWR analysis, tissue volume at ablation temperatures, maximum reached temperatures, and isothermal contours. The double slot (DS) and the monopole (MTM) antennas were chosen to form the arrays, as they were previously designed and tested [17], i.e., their correct performance has been evaluated by experimentation in phantom and porcine ex vivo tissue. Previous experimental tests showed that both antennas could generate ablation temperatures at bone using 10 W of input power applied for durations between 5 and 10 min. Therefore, to avoid tissue overheating, the input power was reduced. In all the analyzed cases, each antenna was fed with 3 W; hence, the treatment time was up to 20 min to generate the largest coverage region. It was observed that the distance between the antennas (*ad*), bone thickness (*bt*), and treatment time play a key role in the antenna array performance. 

The SWR evaluation showed that the DS antenna arrays had a better performance in all the analyzed configurations, while *ad* = 1 mm and *bt* = 25 mm generated the worse-case scenarios. With these dimensions, maximum SWR values of 1.36, 1.95, and 2.15 were found for the linear, triangular, and squared configurations, respectively. In general, the linear configuration shows no problems using antenna distances (*ad*) around 1 mm–15 mm and a bone thickness (*bt*) from 20 mm–40 mm. However, for triangular and square configurations, it was observed that *ad* = 1 mm and *bt* = 25 mm generated the highest SWR values (1.95 and 2.15, respectively), which will make it difficult to work under such conditions because either the patient or the MW equipment can be damaged. On the other hand, the MTM antenna arrays generated higher SWR values than those from the DS antenna arrays. In fact, to implement any configuration of MTM antennas, the bone thickness must be greater than 20 mm, to reduce the SWR values. For a linear configuration, the maximum SWR was 2.24 (*ad* = 1 and *bt* = 30 mm); however, in general, *ad* = 1 mm generated SWR values that were closer to 2. For triangular and squared configurations, the dimension *ad* = 1 mm and *ad* = 5 mm generated SWR between 2.0 and 3.6. Therefore, these cases could be implemented just by adding an impedance coupler to reduce the SWR as much as possible and avoid large power losses that could cause patient overheating as well as some damage to the MW equipment.

The analyzed antenna distance (*ad* = 1, 5, 10, and 15 mm) allows the generation of a continuous ablation zone even after just 1 min of heating by using a total input power of 6 W (linear configuration), 9 W (triangular configuration), and 12 W (square configuration). However, after 1 min of heating, the coverage region is narrow, focused in the area surrounding the slots and monopole; the treatment time must be increased to enhance the thermal ablation effect. After 5 min, the linear configuration of both antennas generated volumes of ablated bone similar to those that were reported by a single antenna under similar conditions [28]. A single DS antenna fed by 5 W applied per 5 min generated 2.4 cm^3^ of ablated bone, while a linear DS antenna array fed by 6 W at the same time generated from 2.69 cm^3^ to 4.65 cm^3^ depending on antenna distances (*ad*) and bone thickness (*bt*). On the other hand, an MTM antenna fed by 5 W applied per 5 min generated an ablation volume of 2.09 cm^3^, while a linear MTM antenna array fed by 6 W at the same time generated from 2.08 cm^3^ to 3.24 cm^3^, also depending on *ad* and *bt*. Moreover, for each configuration, antenna type, and bone thickness (*bt*), a recommended antenna distance (*ad*) was set (See Table 2, Table 4, and Table 6) based on the lowest SWR values, the highest volumes of bone under ablation, and the lowest thermal effect over muscle tissue. As expected, the longer the treatment time, the higher the reached temperatures and the volume of bone under ablation; however, muscle is also affected by the temperature increase. On the other hand, the volume of bone tissue under ablation can also be increased by using either a triangular or a square configuration of any antenna; however, the volume of muscle at ablation temperatures also increases. Therefore, tuning the input power and treatment times could be enough to reduce the thermal effects on muscle. Nevertheless, a cooling system can be implemented if we want to reduce the thermal effect over muscle without modifications on the reached temperatures and volume of ablated bone. Another essential fact to be considered is that the bone thickness is strongly related to the volume of muscle at ablation temperatures, i.e., the thicker the bone, the more negligible effect over the surrounding tissues will be generated. Figure 9 and Figure 11 show the symmetry of the temperature distributions that were generated over the planes XZ and YZ for triangular and square configurations, respectively. Moreover, it is observed how the size of the isothermal contour is modified as a function of treatment time. The linear array does not show symmetry; therefore, Figure 7 shows how the temperature distributions tend to be different at each analyzed plane. 

On the other hand, to our understanding, this study is the first one that was performed to evaluate the heating characteristics of arrays that are composed of micro-coaxial antennas that are specifically designed to treat bone. The DS and the MTM antennas were previously optimized using the finite element method; consequently, their design allows achieving a maximum energy transference. Hence, the required input power to generate thermal ablation can be reduced as much as possible. The evaluation tests that were performed to characterize both antennas showed that 10 W were enough to produce thermal ablation in bone tissue with treatment times around 5–10 min. Therefore, to avoid tissue overheating when antenna arrays are used, each antenna was fed with 3 W, i.e., a total of 6W, 9 W, and 12 W were used to feed the linear, triangular, and square configurations, respectively. These input power levels are far away from those that were reported by other authors. Karampatzakis et al. performed a similar study, implemented by using a triangular and square configuration of DS antennas [23]; they reported 50 W to generate continuous ablation zones with *ad* = 10 mm. Moreover, if the ad increases, for example, *ad* = 15 mm (triangular configuration), at least 75 W is required to generate a continuous ablation zone, while *ad* = 20 mm requires 100 W. Karampatzakis et al. reported some results for ex vivo tissue, they used a triangular array with *ad* = 15 mm and a total input power of 100 W; after 10 min of treatment time, the reported ablation zone dimensions are 5.2 cm × 5.2 cm × 5.0 cm. In the present study, a triangular configuration with 9 W and *ad* = 10 mm and *bt* = 40 mm, after 5 min, generates a T__max_ = 70.26 °C, V__bone_(T > 55 °C) = 7.59 cm^3^, and a coverage region of 2.48 cm × 2.53 cm × 2.50 cm. After 20 min, the same configuration generates T__max_ = 94 °C, V__bone_ (T > 55 °C) = 31.29 cm^3^, and a coverage region of 3.94 cm × 3.94 cm × 3.90 cm. In both cases, muscle is hardly affected. Although the coverage region that was reported by Karampatzakis et al. was larger than the ones that were reported in the present study, in our case, the level of input power that was used was about one-tenth of the reported values. In fact, the distance ad can be increased to increase the coverage region; therefore, the input power could also be increased. Unfortunately, most of the literature reports the evaluation of using antenna array configurations to treat soft tissues [20,21,22,29], making it impossible to compare our results in more detail. As it is reported in the literature, electromagnetic models are maturing rapidly; however, the accuracy of thermal models is still under development. The main problems to achieve accurate models are the uncertainties in tissue thermal properties. These properties are different among tissues, patients, etc. Moreover, these properties are temperature-dependent, i.e., they change while the temperature is changing due to the thermal ablation treatment. Therefore, to develop accurate models to predict temperature distributions is still a challenge. Not only because of the difficulty of modeling tissue properties, but also because of the high computational resources that are required to implement such models. Due to this, our research is a first approach about the performance of multi-antenna arrays to treat bone tumors; constant values of these properties were considered to reduce the complexity of the models and computational resources. However, one of the main goals of this research group is to improve the accuracy of thermal models. Therefore, our next step is to evaluate the performance of these antenna arrays to validate its performance by using phantoms, ex vivo, and in vivo tissue. The information from the experimentation in vivo will help to improve the accuracy of the thermal models. Moreover, the inclusion of a cooling system could help to reduce the thermal effect on muscle tissue. It is important to address that due to the lack of bone tumor properties that are reported in literature, just healthy bone properties were used to implement this study.

## 5. Conclusions

Our main scientific contribution is the evaluation of the thermal performance of different antenna arrays to know the possibility of treating large bone tumors. Moreover, this study is the first one investigating the feasibility of implementing linear, triangular, and square antenna array configurations to treat bone tumors. The linear configuration with DS antennas showed SWR ≤ 1.36 for ad = 1 mm–15 mm and bt = 20 mm–40 mm, while the same configuration with MTM antennas showed SWR ≤ 1.6 for ad = 10 mm–15 mm and bt = 25 mm–40 mm. The triangular configuration for DS and MTM antennas generated SWR ≤ 1.5 for ad = 5 mm–15 mm and bt = 20 mm–40 mm and SWR ≤ 1.5 for ad = 10 mm–15 mm and bt = 25 mm–40 mm, respectively. The square configuration with DS antennas generated SWR ≤ 1.5 for ad = 5 mm–20 mm and bt = 20 mm–40 mm. However, the square configuration with MTM antennas generated SWR ≤ 1.5 just for those cases where ad = 10 mm and bt = 25 mm–40 mm. Therefore, the analyzed antenna arrays could be used under these considerations to produce a larger ablation zone in bone tissue without damaging the surrounding muscle and fat tissue. All the other cases generated higher SWR values (>1.6), which means a lower energy transference from the MW system to the tissue. Moreover, to use the MTM in such configurations, the bone thickness (bt) must be wider than 20 mm. Bone thickness can be directly related to the tumor size where the antenna is inserted. In accordance with the treatment time, and antenna configuration, the T_max and the volume of ablated tissue could be modified, e.g., V_bone = 4.65 cm^3^ and V_bone = 3.24 cm^3^ were obtained with a linear DS and MTM antenna arrays that were fed with a total power of 6 W applied during 5 min, respectively. However, the triangular configuration (9 W) generated V_bone = 7.59 cm^3^ and V_bone = 8.75 cm^3^ with the DS and the MTM antenna arrays, respectively. Moreover, the square configuration (12 W) tends to overheat the tissue, i.e., temperatures higher than 100°C were reached. In the best-case scenarios, V_bone = 10.46 cm^3^ and V_bone = 7.79 cm^3^ were generated by the DS and MTM antenna arrays, respectively. To reduce the maximum temperature, the input power of each antenna can be reduced to less than 3 W. If treatment time increases (20 min), the volume of ablated bone increases; however, muscle tissue is also affected. Nevertheless, this could help to treat a larger region as a safe margin to avoid tumor recurrence. Micro-coaxial antenna arrays could be used to increase the volume of the treated region. Moreover, by using different antenna types and treatment times, different shapes of the ablation zones are generated. Our study provides a wider evaluation of several case scenarios that could happen in the clinic. Therefore, it could help as a guide to choose the antenna type, configuration, input power, treatment time, etc., in accordance with the tumor that is to be treated. To provide a stronger evaluation for future work, modeling including the thermal dependence of tissue properties and blood perfusion must be performed. Moreover, an experimental evaluation must be carried out, either in phantoms, ex, or in vivo tissues.

## Figures and Tables

**Table 1:** Tissues and antenna properties used in the FEM models [26].

Parameter	Value	Parameter	Value
Blood density	1050 (kg/m^3^)	ε_r fat_	10.8 (—)
Specific heat	3639 (J/kg∙K)	σ _fat_	0.268 (S/m)
Blood temperature	37 °C	*k* _fat_	0.21 [W/(m∙K)]
ε_r bone_	18.5 (—)	ρ _fat_	911 [kg/m^3^]
σ _bone_	0.805 (S/m)	*c*_fat_	2348 [J/(kg∙K)]
*k* _bone_	0.31 [W/(m∙K)]	ε_r skin_	38 (—)
ρ _bone_	1908 [kg/m^3^]	σ _skin_	1.46 [S/m]
*c* _bone_	1313 [J/(kg∙K)]	*k* _skin_	0.37 [W/(m∙K)]
ε_r muscle_	52.7 (—)	ρ _skin_	1109 [kg/m^3^]
σ _muscle_	1.74 (S/m)	*c* _skin_	3391 [J/(kg∙K)]
*k*_muscle_	0.49 [W/(m∙K)]	ε_r catheter_	2.6 (—)
ρ _muscle_	1090 [kg/m^3^]	ε_r dielectric_	2.03 (—)
*c* _muscle_	3421 [J/(kg∙K)]		

**Table 2:** Parameters included in the parametric study.

Parameter	Values
Distance between antennas (*ad*)	1, 5, 10, 15 (mm)
Bone thickness (*bt*)	2, 2.5, 3, 3.5, 4 (cm)

**Table 3:** Linear antenna array performance. Best-case scenarios were obtained for each analyzed bone thickness (*bt*) based on the SWR of the antennas. Maximum reached temperature and tissue volume at temperatures higher than 55 °C are presented.

Antenna Array	*ad* [mm]	*bt* [mm]	SWR		T__max_ [°C]		V__bone_ [cm^3^]		V__muscle_ [cm^3^]	
			A_1_	A_2_	5 min	20 min	5 min	20 min	5 min	20 min
**2 double slot antennas (DS)**	5	20	1.05	1.05	85.49	106.10	2.69	9.74	1.36	8.13
	5	25	1.11	1.11	88.23	109.00	3.90	13.00	0.28	4.41
	15	30	1.15	1.15	70	85.22	2.85	13.09	0	2.15
	5	35	1.10	1.10	86.76	109.10	4.67	18.58	0	0.08
	5	40	1.09	1.09	87.41	109.50	4.65	19.15	0	0
**2 monopole antennas (MTM)**	10	25	1.30	1.30	65.60	82.82	2.08	9.81	0.22	5.02
	10	30	1.36	1.36	67.63	84.20	2.71	12.07	0	2.52
	10	35	1.32	1.32	68.7	86.02	3.06	14.83	0	0.48
	10	40	1.29	1.29	68.23	86.01	3.24	16.49	0	0

**Table 4:** Best-case scenarios of ablation zone dimensions for 5 min and 10 min (6 W) for the linear configurations of DS and MTM antennas.

	DS Antenna Array		MTM Antenna Array	
Antenna distance (*ad*) (mm)	15		10	
Bone thickness (*bt*) (mm)	30		40	
Heating time	5 min	20 min	5 min	20 min
D_X_ (cm)	0.71	2.45	1.39	2.77
D_Y_ (cm)	1.91	3.29	2.26	3.76
D_Z_ (cm)	2.66	3.31	1.49	2.80
Sphericity index	0.19	0.73	0.40	0.54

**Table 5:** Triangular antenna array performance. Best-case scenarios were obtained for each analyzed bone thicknesses (*bt*) based on the SWR of the antennas. Maximum reached temperature and tissue volume at temperatures higher than 55 °C are presented.

Antenna Array	*ad* [mm]	*bt* [mm]	SWR			T__max_ [°C]		V__bone_ [cm^3^]		V__muscle_ [cm^3^]	
			A_1`_	A_2_	A_3_	5 min	20 min	5 min	20 min	5 min	20 min
**3 double slot antennas (DS)**	5	20	1.21	1.17	1.24	111.20	139.6	4.83	17.94	2.64	13.55
	10	25	1.30	1.30	1.10	92.05	121.3	6.87	22.92	0.71	10.11
	10	30	1.35	1.35	1.13	71.30	92.48	6.70	23.00	0	7.90
	10	35	1.35	1.35	1.12	70.25	92.62	7.30	28.47	0	2.56
	10	40	1.32	1.32	1.11	70.26	94.53	7.59	31.29	0	0.69
**3 monopole antennas (MTM)**	10	25	1.15	1.15	1.19	85.00	112.1	4.97	19.33	2.44	13.16
	15	30	1.56	1.56	1.52	69.05	91.70	4.92	20.30	0.32	9.52
	10	35	1.26	1.26	1.29	88.31	117.8	7.98	30.13	0	2.12
	10	40	1.17	1.17	1.21	90.20	120.9	8.75	34.91	0	0.30

**Table 6:** Best-case scenarios of ablation zone dimensions for 5 min and 10 min (9 W) for the triangular configurations of DS and MTM antennas.

	DS Antenna Array		MTM Antenna Array	
Antenna distance (*ad*) (mm)	10		10	
Bone thickness (*bt*) (mm)	40		40	
Heating time	5 min	20 min	5 min	20 min
D_X_ (cm)	2.48	3.94	2.27	3.90
D_Y_ (cm)	2.53	3.94	2.84	4.21
D_Z_ (cm)	2.50	3.90	2.29	3.84
Sphericity index	0.96	0.98	0.64	0.84

**Table 7:** Square antenna array performance. Best-case scenarios were obtained for each analyzed bone thickness (*bt*) based on the SWR of the antennas. Maximum reached temperature and tissue volume at temperatures higher than 55 °C are presented.

Antenna Array	*ad* [mm]	*bt* [mm]	SWR				T__max_ [°C]		V__bone_ [cm^3^]		V__muscle_ [cm^3^]	
			A_1_	A_2_	A_3_	A_4_	5 min	20 min	5 min	20 min	5 min	20 min
**4 double slot antennas**	5	20	1.21	1.21	1.20	1.21	132.3	139.9	7.15	26.22	4.30	20.79
	15	25	1.36	1.36	1.36	1.36	67.45	88.30	6.86	27.03	0.52	20.00
	15	30	1.32	1.35	1.36	1.35	67.83	91.44	9.04	33.63	0	14.36
	15	35	1.32	1.44	1.45	1.45	66.61	92.53	9.86	40.56	0	7.31
	15	40	1.25	1.43	1.44	1.44	67.03	93.7	10.46	44.53	0	3.86
**4 monopole antennas**	10	25	1.09	1.07	1.07	1.09	105.3	140	7.79	27.69	4.24312	17.51
	10	30	1.04	1.04	1.04	1.03	110.4	140	10.17	34.09	2.11448	13.21
	10	35	1.02	1.03	1.03	1.02	109.2	140	12.28	40.85	0.42405	7.75
	10	40	1.04	1.03	1.03	1.04	108.1	140	13.56	46.41	0	3.10

**Table 8:** Best-case scenarios of ablation zone dimensions for 5 min and 20 min (12 W) for the square configurations of DS and MTM antennas.

	DS Antenna Array		MTM Antenna Array	
Antenna distance (*ad*) (mm)	15		10	
Bone thickness (*bt*) (mm)	40		25	
Heating time	5 min	20 min	5 min	20 min
D_X_ (cm)	2.074	4.19	2.58	—
D_Y_ (cm)	2.68	5.06	4.14	—
D_Z_ (cm)	2.06	4.15	2.56	—
Sphericity index	0.59	0.67	1.5	

## Data Availability

Data available on request from the authors.
